# Development of a fermented quinoa beverage with autochthonous lactic acid bacteria

**DOI:** 10.3389/fmicb.2025.1736226

**Published:** 2026-01-29

**Authors:** Luisina Fontana, Guillermo H. Peralta, Carina Bergamini, María Victoria Beret, Soledad Caballero, Analía Ale, Giuliano Nicola, Liliana Forzani, Gabriel Vinderola, Melisa Puntillo

**Affiliations:** 1 Instituto de Lactología Industrial (CONICET-UNL), Universidad Nacional del Litoral, Santa Fe, Argentina; 2 Cátedra de Toxicología, Farmacología y Bioquímica Legal, Facultad de Bioquímica y Ciencias Biológicas, Universidad Nacional del Litoral, CONICET, Santa Fe, Argentina; 3 Departamento de Matemática, Facultad de Ingeniería Química, Universidad Nacional del Litoral and Instituto de Matemática Aplicada del Litoral (CONICET-UNL), Santa Fe, Argentina

**Keywords:** fermentation, food, lactic acid bacteria, *Lactobacillus*, quinoa

## Abstract

**Introduction:**

The growing demand for plant-based functional foods has driven research into non-dairy fermented alternatives that can deliver live microorganisms and potential health benefits. The pseudocereal Quinoa is a substrate of interest for lactic acid fermentation. This study aimed to develop a fermented quinoa-based beverage using autochthonous lactic acid bacteria (LAB) strains with technological and functional potential.

**Methods:**

Six LAB strains previously isolated from plant sources were screened for growth kinetics in an animal-free medium and in quinoa extract (QE). *Lactiplantibacillus plantarum* LpAv and *Limosilactobacillus fermentum* Lf2, an exopolysaccharide (EPS)-producing strain, were selected for beverage development. Fermentation parameters, rheological and biochemical profiles, peptide release, and sensory attributes were evaluated. An animal trial assessed immunomodulatory and antioxidant capacity in BALB/c mice that received fermented QE.

**Results:**

Both strains were able to acidify QE to pH < 4.5 within 8 h, ensuring microbiological safety. EPS production by Lf2 improved viscosity and texture, while mixed fermentation enhanced lactic acid yield and impacted on peptidic profiles, indicating synergistic proteolytic activity. LAB remained viable (>8 log CFU/mL) after 28 days at 4 °C. Sensory testing (*n* = 111 participants) showed moderate acceptability, improved by artificial flavoring. In mice, fermented QE increased intestinal IL-10 and IFN-γ levels and elevated hepatic catalase and superoxide dismutase activities, suggesting antioxidant and immune-modulatory effects without bacterial translocation.

**Conclusion:**

This work demonstrates the feasibility of producing a safe, stable, and functionally active fermented quinoa beverage using locally sourced LAB. The combination of *L. plantarum* LpAv and *L. fermentum* Lf2 improved both technological and functional properties, supporting their potential as starter cultures for plant-based probiotic foods.

## 1 Introduction

In recent years a growing awareness about the link between diet and health mediated by the gut microbiome has driven the increasing demand for innovative food products targeting it (Deziderio et al., 2023). In response, the food industry has increased its focus on the development of functional foods, food products that provide health benefits beyond the provision of basic nutrients (Rai et al., 2019). Among functional foods, those fermented by lactic acid bacteria (LAB) have received particular attention due to their positive effects on nutritional quality, sensory attributes, and shelf life. LAB are strict fermenters that primarily convert simple carbohydrates into lactic acid, and their ability to grow in acidic environments (pH < 5) gives them a competitive advantage in low-pH niches. LAB are microorganisms Generally Regarded as Safe (GRAS status by the US Food and Drug Administration), with many species included in the EFSA’s Qualified Presumption of Safety (QPS) list (Leroy and De Vuyst, 2004).

Historically dairy products such as yogurt and milk kefir have served as common carriers for beneficial bacteria, but there is simultaneously an increasing interest in plant-based alternatives (Kandylis et al., 2016). These food products meet the increasing consumer demand for vegetarian, vegan, and allergen-free options while offering nutritional and functional benefits (Hidalgo-Fuentes et al., 2024). Plant-derived substrates, such as cereals, pseudocereals, legumes, nuts, and seeds, contain diverse nutrients and can be potentially fermented to improve both their health-promoting properties and sensory qualities (Dahiya and Nigam, 2022). Quinoa (*Chenopodium quinoa*) is a pseudocereal native to the Andean region in Latin America, characterized by a high-quality protein profile that includes all essential amino acids. Quinoa is rich in minerals (Ca, P, Fe, Zn) and vitamins (B, C, E), and various bioactive compounds (Abdelshafy et al., 2024). Quinoa has potential as a fermentative substrate, showing compatibility with LAB fermentation, especially with strains of the species *Lactiplantibacillus plantarum*, supporting bacterial viability after fermentation and storage. In addition, fermentation of quinoa can reduce anti-nutritional compounds like saponins, tannins, and phytic acid, while enhancing its antioxidant, anti-inflammatory, antidiabetic, and anticancer potential (Huang et al., 2022).

Developing plant-based fermented products presents technological challenges. In particular, plant proteins often form weaker, less cohesive gels than dairy proteins as caseins, which can lead to phase separation and reduced viscosity in the fermented product (Zannini et al., 2018). These issues may be mitigated by using LAB strains capable of producing exopolysaccharides (EPS), or by adding natural stabilizers such as pectins, gums, or starches. In addition, rapid acidification is essential for food safety, as slow pH drops may allow the growth of harmful microorganisms like *Bacillus cereus*, making the choice of starter cultures a key consideration in the development of a fermented food (Tetra Pak International S.A., 2021).

Beyond these technological aspects, fermented foods are also gaining attention for their positive effects on gut microbiota, which plays a central role in immune, metabolic, and neurological health. Diet deeply influences microbiota composition: fiber-rich foods promote beneficial microbial populations, and fermented foods contribute to microbial diversity by introducing live microorganisms (Marco et al., 2022). Growing evidence links regular consumption of fermented foods with improved metabolic markers, reduced risk of type 2 diabetes and cardiovascular disease, and better gastrointestinal and immune function. In consequence, some researchers support the incorporation of foods containing live microorganisms into national dietary guidelines (Iyer et al., 2024). It is important to note that although the terms “fermented food” and “probiotic” are often used interchangeably, only strains that meet specific criteria—such as proper identification, viability, and scientifically demonstrated health effects—should be classified as probiotics (Hill et al., 2014). This study aimed at developing a prototype of a plant-based fermented beverage using quinoa as a substrate and selected autochthonous LAB strains with probiotic potential.

## 2 Materials and methods

### 2.1 Lactic acid bacteria

#### 2.1.1 Lactic acid bacteria strains

A total of six LAB strains belonging to the culture collection of the INLAIN (CONICET-UNL) were used in this study: *Lactiplantibacillus plantarum* LpAv and LpS13, *Lacticaseibacillus paracasei* LcAv and LpaM1, *Lacticaseibacillus rhamnosus* LrM1 and *Limosilactobacillus fermentum* Lf2. The first five strains were isolated from different forages: LpAv and LcAv from oat, LpS13 from sorghum, LrM1 and LpaM1 from maize (Puntillo et al., 2020). *L. fermentum* Lf2, an EPS-producing strain, was isolated from cream cheese that presented technological defects (Ale et al., 2016). Strains are kept frozen at −70 °C in the INLAIN culture collection. Before use, the strains were cultured twice in 10 mL MRS broth (Biokar, Beauvais, France) at 37 °C in aerobiosis.

#### 2.1.2 Growth kinetics of LAB strains in MRS and MRSv

MRS and plant-based MRS (MRSv) broth were prepared from single ingredients (obtained from Cicarelli and Microquim, Santa Fe, Argentina), following the composition of commercial MRS (Biokar, Beauvais, France) but replacing meat peptone by the same amount of soy peptone (Microquim, Santa Fe, Argentina) in MRSv. Culture media were sterilized in autoclave (121 °C, 15 min). Growth kinetics were obtained as previously described by (Puntillo et al., 2020). Briefly, each overnight culture of the strains under study in MRS and MRSv broth (10^9^ cfu/mL) was washed twice and diluted 1:10 in sterile Phosphate-Buffered Saline Solution (PBS, pH 7.4). Then, the suspensions were inoculated 1% (v/v) in the corresponding culture medium, at a concentration of ca. 10^6^ cfu/mL and distributed in 96-well microplates (300 μL/well). The plate was incubated at 37 °C in aerobiosis and optical density (OD_570nm_) was measured every 30 min in a Microplate Photometer (Thermo Scientific Multiskan FC). Each strain was assayed in independent triplicates, and the results were expressed as log_10_ cfu/mL. The kinetic parameters *μ*_max_ (maximum growth rate as variation of OD in time, in h) and *λ* (lag phase, in h) were obtained using the DMFit add-in version 3.5 (Institute of Food Research, Norwich Research Park) of Microsoft Excel, fitting the curves obtained to the modified Gompertz model according to Zwietering et al. (1990).

#### 2.1.3 Freeze drying of LAB strains and survival along storage

A batch culture of each strain was obtained in MRSv (450 mL, 37 °C, 18 h, aerobiosis), the biomass was harvested by centrifugation (6,000 × *g*, 15 min, 8 °C), washed twice with sterile PBS buffer (pH 7.4) and resuspended in 160 mL of 15% (w/v) maltodextrin solution. Cell suspensions were distributed in sterile glass cryovials (2 mL/cryovial) and frozen (−70 °C, 24 h). The freeze-drying process was carried out at −55 °C and 0.0010 mBar for 22 h, using an Alpha 1–4 LD plus freeze-dryer (Christ, Germany). Freeze-dried cultures were sealed under vacuum and maintained at 4 and 22 °C. Counts of LAB were performed periodically for 12 months in independent triplicates.

### 2.2 Production of a prototype of fermented quinoa

#### 2.2.1 Preparation of quinoa extract

Quinoa (*Chenopodium quinoa*) seeds, obtained from a local store, were washed with running tap water to remove saponins and impurities, hydrated in tap water (12 h at 4 °C), boiled (15 min) and filtered (cloth filter). Hydrated quinoa seeds were resuspended (12.5% w/v) in clean tap water and processed in a domestic blender at maximum speed until a homogeneous mixture was obtained. The suspension was filtered (cloth filter) and the liquid fraction (quinoa extract, QE) was recovered and autoclaved (121 °C, 15 min) to obtain a sterile QE.

#### 2.2.2 Growth kinetics in quinoa

Overnight cultures (broth MRSv, 37 °C, aerobiosis, 18 h) of each strain were washed twice with sterile phosphate buffered saline (PBS, pH 7.4), resuspended in PBS to the original volume, and inoculated (1% v/v) in 5 mL of QE. Fermentation was carried out at 37 °C and the pH and LAB counts (MRS agar, 37 °C, 48 h, aerobiosis) were determined at the beginning and after 2, 4, 6, 8, 10 and 24 h of fermentation. Fermentations were performed in independent triplicates.

### 2.3 Quinoa fermentation with selected LAB

#### 2.3.1 Use of sucrose for EPS synthesis

In order to induce EPS synthesis by *L. fermentum* Lf2, increasing concentrations of sucrose (2, 4, 5 and 10% w/v) were added to QE before fermentation (Ale et al., 2019). Based on sensory perceptions of explorative assays (data not shown), LAB counts and pH values, the study was continued using 2 and 4% (w/v) of sucrose, for which growth and pH kinetics of QE inoculated with *L. fermentum* Lf2, *L. plantarum* LpAv and a mix of both strains were performed. To obtain the mix, the cultures of both strains were mixed in a 1:1 ratio and inoculated at 1% (w/v). Fermentation was carried out at 37 °C, until pH value was lower than 4,5, in independent triplicates.

#### 2.3.2 Rheological characterization of fermented QE

To study the rheological behavior of QE fermented with *L. plantarum* LpAv, *L. fermentum* Lf2 and the mixture of both strains, flow curves (shear stress -*σ*, Pa- vs. shear rate -*γ*, s − 1) and dynamic viscosity (*μ*) were obtained by rotational rheometry with parallel plate geometry, 1 mm gap, at controlled temperature (7.0 ± 0.5 °C) (HAAKE MARS 40, Thermo Scientific, United States). Sweeps were performed in the range of γ:0–300 s^−1^ (Zannini et al., 2018) with modifications. Analyses were performed in independent duplicate. Results were analyzed by one-way ANOVA and Tukey’s test for comparison of means (significance value: *p* < 0.05).

#### 2.3.3 Chemical analysis of fermented QE

Lactic acid, ethanol and sucrose contents were determined by HPLC (Knauer Smartline, Berlin, Germany) in the supernatant of fermented QE, obtained by precipitation with 0.6 mmol/L trichloroacetic acid and centrifugation (17,000 × *g*, 20 min, 4 °C). Samples were filtered (0.45 μm filters) and 20 μL aliquots were injected onto an Aminex HPX-87H ion-exclusion column. Separation was performed by chromatography under the following conditions: isocratic mobile phase, H_2_SO_4_ (5 mmol/L); flow rate, 0.6 mL/min; and column temperature, 45 °C. For data analysis, a refractive index detector (Knauer Smartline) connected to the software (Peak Simple II) was used.

#### 2.3.4 Peptidic profile

The peptidic profile analysis in fermented QE was determined by high-performance liquid chromatography (HPLC). Samples were acidified to pH 4.0 with 2 N hydrochloric acid and allowed to stand at room temperature for 15 min. The acidified samples were subsequently centrifuged at 14,000 × *g* for 30 min at 20 °C. The resulting supernatant was filtered through fast-flow filter paper and subsequently diluted 1:10 (v/v) with Solvent A, consisting of 0.11% (v/v) trifluoroacetic acid in distilled water. After resting 15-min resting period, the dilution was centrifuged at 17,000 × *g* for 20 min, and the supernatant was filtered through a 0.2 μm nylon membrane filter prior to HPLC injection. Chromatographic separation was performed according to the method of Dunand et al. (2019), with minor modifications. An Aquapore OD-300 C18 analytical column (220 mm × 4.6 mm) was used at 30 °C, with a flow rate of 0.9 mL/min. Eluted compounds were detected at 214 nm using a UV–Vis detector. Data acquisition and processing were performed using Chromera software (Perkin Elmer). The column was initially equilibrated with 100% Solvent A, and 60 μL of the sample was injected under these conditions. Five minutes after injection, a gradient was applied, increasing the concentration of Solvent B (0.1% TFA in acetonitrile:water, 60:40) from 0 to 100% over 25 min. The final conditions (100% Solvent B) were maintained for 5 min. The column was then returned to initial conditions (100% Solvent A) over 10 min and re-equilibrated for an additional 10 min prior to the next injection.

#### 2.3.5 Survival of LAB along storage

Cell counts of LAB and pH measurements were performed on fermented QE immediately after fermentation and at days 7, 14, 21, and 28 of storage at 4 °C. Counts were performed in independent triplicates.

#### 2.3.6 Consumer-based sensory analysis

To evaluate the acceptability of the beverages under study, an overall acceptability test was conducted using a 9-point hedonic scale, ranging from “like extremely” to “dislike extremely” (Ruiz-Capillas et al., 2021). The experiment was carried out at the Faculty of Chemical Engineering (National University of the Litoral), in the Food Sensory Analysis Laboratory, a facility specifically designed for sensory testing. For the evaluation, two fermented QE samples were prepared using the mixed strains, to which food-grade colorants and flavorings (vanilla and strawberry) were added to improve appearance and flavor (Fleibor S. R. L. Laboratory, Buenos Aires, Argentina), following the manufacturer’s instructions. Each sample was placed in individual transparent plastic containers (55 mL) with lids, filled to 50% of their capacity, and coded with randomly assigned three-digit numbers. Samples were stored under refrigeration (4 °C) until the time of evaluation. The presentation order of the samples was randomized for each panelist to minimize bias.

### 2.4 Animal trial

#### 2.4.1 Animals and feeding procedures

A total of 50 male BALB/c mice (6-week-old) were obtained from the random inbred colony of the Institute of Veterinary Sciences from Litoral (ICiVet-Litoral, UNL-CONICET), Veterinary Sciences Faculty, National University of Litoral (Esperanza, Santa Fe, Argentina). Animals were allowed to stand at the INLAIN (CONICET-UNL) animal facility for 7 days before starting the animal treatments. The 3Rs principle was considered. Mice were maintained and treated according to the guidelines of the National Institute of Health (NIH, Bethesda, MD, United States). Each experimental group consisted of 10 mice housed in groups of five, in 2 plastic cages per group kept under controlled environmental conditions (21 ± 1 °C, 55 ± 2% humidity, 12 h light/dark cycle, 20 renovations of the air volume of the room by hour). Five groups were set: (1) control group (CW) that received sterile 0.3 mL of tap water by gavage, (2) control group (C-QE) that received 0.3 mL of QE by gavage, (3) Lf2 group (Lf2-QE) that received QE fermented with *L. fermentum* Lf2, (4) LpAv group (LpAv-QE) that received QE fermented with *L. plantarum* LpAv and (5) mix group (Mix-QE) that received QE fermented with the mix of strains. Mice of groups 3, 4 and 5 received daily (by gavage) 0.3 mL of fermented QE at a LAB concentration of ca. 7.5 log cfu/mL. The period of 15 days was chosen according to preliminar results obtained in our lab. All animals received, simultaneously and *ad libitum*, sterile tap water and a conventional balanced diet: proteins 230 g/kg, raw fiber 60 g/kg, total minerals 100 g/kg, Ca 13 g/kg, P 8 g/kg, water 120 g/kg and vitamins (Cooperación, Buenos Aires, Argentina). Animal procedures were approved by the Experimental Work Safety and Ethics Committee of the CCT-CONICET (Santa Fe) CEYSTE-CES-01260/2023.

#### 2.4.2 Translocation assay

At the end of the feeding period (15 days) mice were intraperitoneally anesthetized with 0.3 mL of a cocktail [(xylazine (20 mg/mL) 9 parts + ketamine (100 mg/mL) 9 parts + acepromazine (10 mg/mL) 3 parts + sterile saline solution 79 parts)]. A portion of liver was aseptically removed, homogenized in sterile PBS buffer and plated (1 mL) in Violet Red Bile Lactose (VRBL) agar (Biokar, Beauvais, France) for detection of enterobacteria. Plates were incubated at 37 °C for 24 h under aerobic conditions. Results were expressed as positive or negative translocation since the liver is a sterile organ under normal conditions.

#### 2.4.3 Immunomodulation potential of fermented quinoa extract

The small intestine content was flushed with 5 mL of cold PBS containing a protease inhibitor cocktail (Sigma Aldrich, St. Louis, MO, United States). The intestinal fluid was centrifuged (2,000 × *g*, 15 min, 4 °C) and the supernatant was stored at −70 °C for secretory-IgA quantification by ELISA (Ale et al., 2016). Samples of small and large intestines were recovered and kept frozen (−70 °C) for cytokine determination. Intestine samples were homogenized (Ultra Turrax T8, Ika Labortechnik, Staufen, Germany) in PBS solution containing 1% (v/v) antiprotease cocktail (Sigma), 10 mmol/L EDTA (Sigma) and 0.05% (v/v) Tween 20 (Sigma) in a proportion of 1 mL PBS:100 mg tissue. The samples were then centrifuged (10 min, 10,000 × g, 4 °C) and the supernatant was collected and kept frozen for cytokine quantification. The concentration of IL-10, IL-12, IL-6, TNF-*α* and IFN-*γ* was measured by ELISA using commercially available antibodies (BD Biosciences Pharmingen, San Diego, CA, United States), according to the procedures supplied by the manufacturer.

#### 2.4.4 Oxidative stress and enzyme tissue damage in liver

Portions of livers were kept frozen at −80 °C until further analysis. The extracts for measuring the activities of antioxidant enzymes were prepared by liver homogenization with a potassium chloride buffer (0.15 M) containing benzamidine (0.2 mM) and phenylmethylsulfonyl fluoride (PMSF, 0.5 mM), according to Sabatini et al. (2011). The activity of superoxide dismutase (SOD) was determined by its ability to inhibit epinephrine autoxidation according to Misra and Fridovich (1972), and catalase (CAT) activity was measured through the decomposition of H2O2 following the procedure by Aebi (1984) with modifications proposed by Poletta et al. (2016). In addition, lipid peroxidation levels (LPO) were measured from the same extracts following the method proposed by Buege and Aust (1978) based on thiobarbituric acid reactive substances (TBARS) assay: an aliquot of each homogenization was mixed with trichloroacetic acid 15% (w/v) (TCA), thiobarbituric acid (TBA) 0.375% (w/v), and chloride acid (0.25 M), and then butylated hydroxytoluene (BHT) 4% was added. The mixture was heated in a dry bath at 95 °C for 45 min and, after cooling, the precipitated fraction was removed by centrifugation (6,700 × *g*, 10 min). From the same homogenates, enzyme tissue damage was measured through the activities of alkaline phosphatase (ALP), glutamic oxaloacetic transaminase (GOT), and glutamate-pyruvate transaminase (GPT) by employing commercial kits (Wiener Lab^®^). Each sample was measured in triplicate, and all the markers were expressed in terms of protein content determined with a commercial kit (Proti U/LCR, Wiener Lab^®^).

#### 2.4.5 Determination of organic acids in feces

Acetic, propionic, butyric and lactic acid were determined by HPLC (Ale et al., 2024). In brief, samples were resuspended in 0.01 M H_2_SO_4_ at a 1:10 ratio (mobile phase) and centrifuged at 10,000 × *g* for 10 min. The resulting supernatant was adjusted to pH 2 by adding a fixed volume of H_2_SO_4_ 2 M and incubated at 65 °C for 20 min. After a second centrifugation at 16,000 × *g* for 30 min, the supernatant was filtered through 0.45 μm membranes (Millipore, São Paulo, Brazil) and injected into the HPLC system. The chromatographic system (Perkin Elmer, Norwalk, CT, United States) included a quaternary pump, an online degasser, a column oven, and two in-line detectors: a UV–visible detector set at 210 nm and a refractive index (RI) detector maintained at 35 °C. The column oven and RI detector belonged to the Flexar Series, while the remaining components were from the 200 Series. Organic acids were detected by both detectors, but quantification was performed using only the RI detector. Isocratic elution was carried out at 65 °C using 0.01 M H₂SO₄ as the mobile phase at a flow rate of 0.6 mL/min, with an Aminex HPX-87H column (300 × 7.8 mm) coupled to a H + cation microguard cartridge (Bio-Rad Laboratories, Hercules, CA, United States). Calibration curves were prepared using analytical-grade standards (Sigma Aldrich, United States).

### 2.5 Statistical analysis

Microbiological and physicochemical determinations were performed in triplicate and the results were presented as mean ± standard deviation. One-way ANOVA procedure of SPSS 15.0 software (SPSS Inc., Chicago, IL, United States) was used to data analyses. Tukey and Duncan’s Multiple Range test were used to detect significant differences between means of the control and fermented quinoa. Differences were considered statistically significantly different when *p* < 0.05. The statistical analysis of the animal trial was performed using SigmaPlot 12.0 (Systat Software Inc., San Jose, CA, United States). A linear mixed-effects (LME) model with random effects for individual mice was applied, followed by Dunnett’s *post hoc* test to evaluate differences between the control and experimental groups.

## 3 Results

### 3.1 Growth kinetics in broths

A culture broth free of animal components (MRSv) was prepared *in house* by replacing meat peptone in MRS by soy peptone, and the ability of the LAB strains to growth in this broth was evaluated and compared to MRS. The kinetic growth parameters (*μ*_max_ and *λ*) for *L. plantarum* LpAv and LpS13, *L. paracasei* LcAv and LpaM1, *L. rhamnosus* LrM1 and *L. fermentum* Lf2 were determined in MRS and MRSv (Table 1). The *μ*_max_ values significantly decreased (*p* < 0.05) when the strains were cultured in MRSv compared to MRS, with the exception of *L. plantarum* LpAv, which did not exhibit a significant change (*p* > 0.05). Specifically, the μ_max_ values of *L. paracasei* LpaM1 decreased approximately 2.5-fold (*p* < 0.05). Furthermore, the λ values were 1.3 to 2.6 times lower (*p* < 0.05) for *L. paracasei* LcAv and LpaM1, and *L. plantarum* LpAv and LpS13 in MRSv compared to MRS. Conversely, *L. fermentum* Lf2 showed a significantly higher λ value in MRSv (*p* < 0.05).

**Table 1:** Kinetic parameters (*μ*_max_ and *λ*) of the growth of *L. rhamnosus* LrM1, *L. plantarum* LpS3, *L. fermentum* Lf2, *L. paracasei* LcAv, *L. paracasei* LpaM1, and *L. plantarum* LpAv in MRS and MRSv media.

Medium	Strain	*μ*_max_	*λ*	*R* ^2^
MRS	LrM1	0.24 ± 0.01^a^	0.79 ± 0.01^a^	0.986
	LpS3	0.41 ± 0.01^a^	6.78 ± 0.02^a^	0.980
	Lf2	0.22 ± 0.01^a^	2.15 ± 0.01^a^	0.997
	LcAv	0.18 ± 0.03^a^	1.53 ± 0.17^a^	0.996
	LpaM	0.54 ± 0.01^a^	6.74 ± 0.01^a^	0.984
	LpAv	0.25 ± 0.02^a^	6.03 ± 0.01^a^	0.988
MRSv	LrM1	0.20 ± 0.01^b^	0.57 ± 0.03^a^	0.986
	LpS3	0.26 ± 0.01^b^	3.13 ± 0.18^b^	0.998
	Lf2	0.20 ± 0.01^b^	3.09 ± 0.13^b^	1.000
	LcAv	0.11 ± 0.01^b^	0.72 ± 0.11^b^	0.993
	LpaM	0.22 ± 0.02^b^	5.12 ± 0.22^b^	0.988
	LpAv	0.23 ± 0.01^a^	2.30 ± 0.14^b^	0.991

^a,b^Values with different superscripts differ significantly (*p* < 0.05) for each strain between culture media. Values are the means (±SD) of three replicates.

### 3.2 Stability of freeze-dried cultures along storage

**Figure 1:**
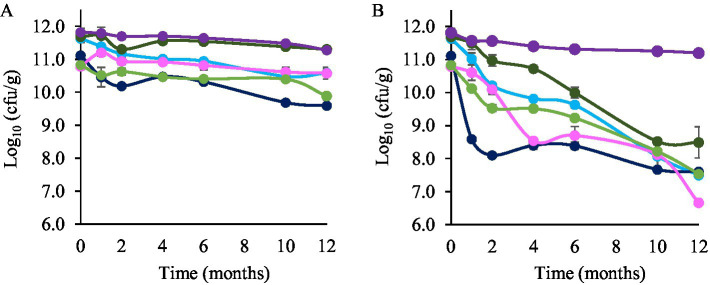
Bacterial counts (log_10_ cfu/g) of freeze-dried *L. paracasei* LcAv (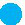), *L. plantarum* LpAv (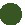), *L. fermentum* Lf2 (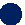), *L. rhamnosus* LrM1 (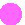), *L. plantarum* LpS3 (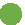), and *L. paracasei* LpaM1 (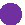), stored at 4 °C **(A)** and 22 °C **(B)**. Values are means (±SD) of three repetitions.

Maltodextrin was used as a cryoprotectant. The percentage of survival to the freeze-drying process was 98.4% for *L. plantarum* LpAv, 95.9% for *L. fermentum* Lf2, 93.4% for *L. rhamnosus* LrM1, 94,9% for *L. paracasei* LpaM1, 94.4% for *L. plantarum* LpS3 and 100% for *L. paracasei* LcAv. The counts of the strains immediately after freeze-drying were 11.81 ± 0.14, 11.12 ± 0.06, 10.79 ± 0.10, 11.68 ± 0.16, 10.83 ± 0.04 and 11.63 ± 0.13 log cfu/g for *L. plantarum* LpAv, *L. fermentum* Lf2, *L. rhamnosus* LrM1, *L. paracasei* LpaM1, *L. plantarum* LpS3 and *L. paracasei* LcAv, respectively. Counts of freeze-dried LAB cultures were periodically assessed along 12 months of storage (Figure 1). Bacterial counts of *L. rhamnosus* LrM1 and *L. paracasei* LpaM1 showed no significant differences (*p* > 0.05) at 4 °C during the studied period, indicating a high survival rate for both strains. The counts of *L. plantarum* LpAv significantly decreased (*p* < 0.05) by 0.5 log units after 12 months of storage, maintaining the same order of magnitude and remaining at a satisfactory level at the end of the assay. *L. paracasei* LcAv and *L. plantarum* LpS3 viability decreased ca. 0.9 and 1 log orders, respectively. *L. fermentum* Lf2 was the least resistant strain, with a decrease of 1.52 log units after 12 months at 4 °C, with a final count of 9.60 ± 0.10 log (cfu/g).

Regarding storage at 22 °C, the bacterial counts of *L. plantarum* LpS3, *L. paracasei* LcAv, and *L. fermentum* Lf2 significantly decreased after 1 month. Notably, *L. fermentum* Lf2 exhibited the most substantial viability loss, with a decrease of 2.53 log orders compared to its initial count. Additionally, after 2 months, the bacterial counts of all of the strains (except *L. plantarum* LpAv) showed significant differences when compared to the initial counts. In particular, *L. rhamnosus* LrM1 showed the lowest survival rate at 22 °C, with a decrease of 4.13 log units after 12 months. In contrast, *L. plantarum* LpAv was the most resistant strain, with a reduction in cell counts of ca 0.61 log units (*p* < 0.05) over the same period.

### 3.3 Fermentation of QE with different LAB strains

**Figure 2:**
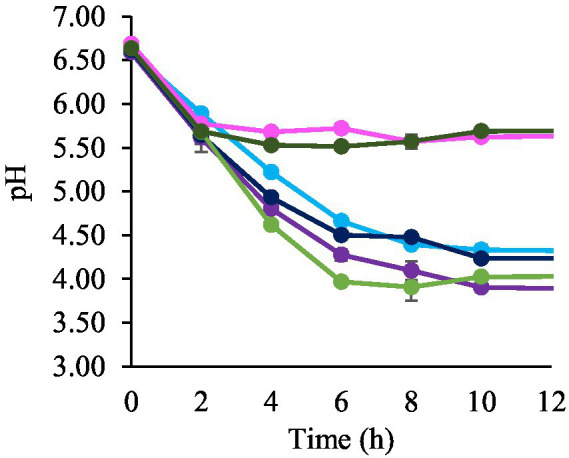
pH values of QE inoculated with *L. paracasei* LcAv (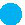), *L. plantarum* LpAv (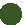), *L. fermentum* Lf2 (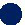), *L. rhamnosus* LrM1 (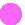), *L. plantarum* LpS3 (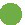), and *L. paracasei* LpaM1 (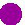), at the beginning (0) and after 2, 4, 6, 8, 10 h of fermentation. Values are means (±SD) of three repetitions.

A visually homogeneous QE was obtained, with a pH of 6.66 ± 0.10 and satisfactory consistency for the subsequent fermentation assays. To select the most promising LAB for developing a plant-based beverage prototype, an assay was conducted to evaluate the fermentative capacity of the six strains under study in a quinoa-based beverage. The pH curves of the fermented QE are shown in Figure 2. After 2 h of incubation, all the strains significantly decreased pH, with values ranging from 5.62 to 5.89. Notably, the pH of QE inoculated with *L. paracasei* LpaM1 and *L. rhamnosus* LrM1 exhibited no significant changes (*p* > 0.05) from 4 h until the end of the fermentation. On the other hand, *L. fermentum* Lf2, *L. plantarum* LpAv and LpS3 lowered pH below 4.5 after 6 h of fermentation, with values of 4.50 ± 0.05, 4.28 ± 0.06, and 3.97 ± 0.01, respectively, significantly differents (*p* < 0.05) among them. After 10 h of fermentation, the lowest pH were obtained with *L. plantarum* LpAv (3.90 ± 0.01) and LpS3 (4.02 ± 0.01), without significant differences between them.

**Figure 3:**
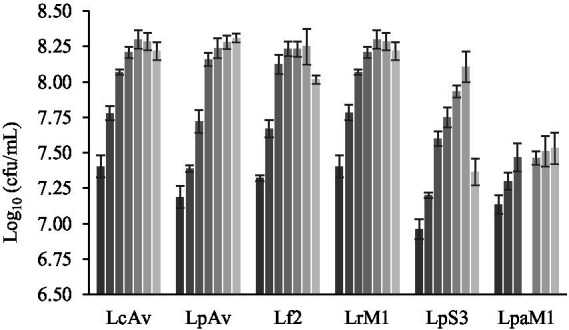
Total LAB count (Log_10_ cfu/mL) of EQ fermented by *L. paracasei* LcAv, *L. plantarum* LpAv, *L. fermentum* Lf2, *L. rhamnosus* LrM1, *L. plantarum* LpS3, and *L. paracasei* LpaM1, at the beginning (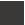) and after 2 (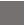), 4 (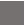), 6 (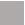), 8 (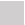), 10 (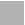) h of fermentation. Values are means (±SD) of three repetitions.

Regarding the growth of LAB in QE during the fermentation (Figure 3), *L. plantarum* LpAv, *L. paracasei* LcAv, *L. rhamnosus* LrM1, and *L. fermentum* Lf2 displayed a growth of 0.81–0.97 log orders with respect to the inoculated level. These counts (higher than 8 log_10_ cfu/mL) remained stable, for the first three strains, until the end of fermentation (24 h). On the other hand, the counts of *L. fermentum* Lf2 and *L. plantarum* LpS3 decreased significantly to 8.02 ± 0.03 and 7.36 ± 0.09, respectively, between 10 and 24 h of fermentation.

### 3.4 Fermentation of QE with *L. plantarum* LpAv and *L. fermentum* Lf2

#### 3.4.1 LAB counts and pH values

**Figure 4:**
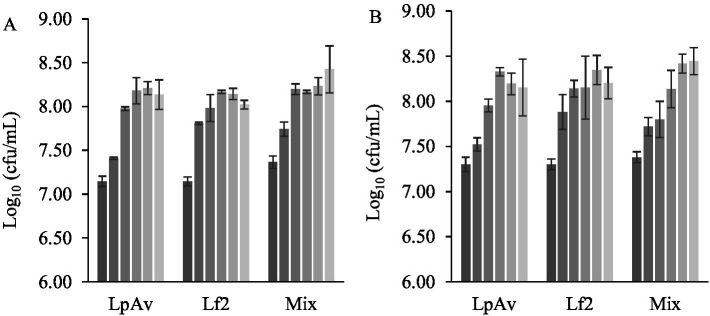
Total LAB count (Log_10_ cfu/mL) of EQ, with the addition of 2% **(A)** and 4% **(B)** (w/v) of sucrose, fermented by *L. plantarum* LpAv, *L. fermentum* Lf2, and *L. plantarum* LpAv + *L. fermentum* Lf2 (Mix), at the beginning (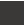) and after 2 (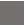), 4 (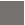), 6 (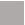), 8 (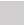), 10 h (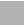) of fermentation. Values are means (±SD) of three repetitions.

**Figure 5:**
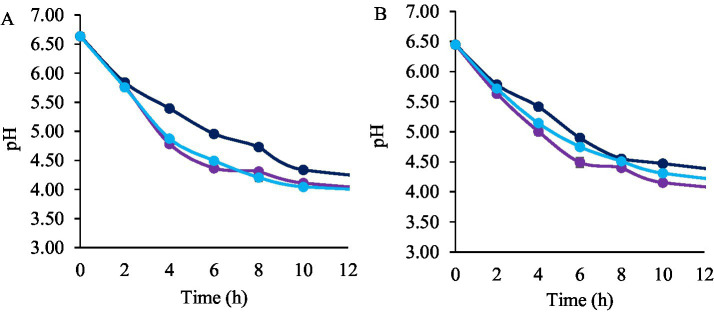
pH values of the QE with the addition of 2 **(A)** and 4 **(B)** % (w/v) of sucrose, inoculated with *L. plantarum* LpAv (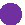), *L. fermentum* Lf2 (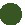) and a mix (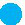) of *L. plantarum* LpAv + *L. fermentum* Lf2, at the beginning and after 2, 4, 6, 8 and 10 h of fermentation. Values are means (±SD) of three repetitions.

*Lactiplantibacillus plantarum* LpAv and *L. fermentum* Lf2 were selected due to their satisfactory acidification capacity and LAB counts compared to the other strains. LpAv also had displayed immunomodulatory capacity in a previous study (Puntillo et al., 2021), while Lf2 is an EPS-producing strain with interesting technological and functional capacities (Ale, et al., 2019). Recent studies reported that yogurts produced with Lf2 enhanced the release of bioactive peptides and significantly modified organic acid and carbohydrate levels, as well as rheological properties and microstructure (Ale et al., 2024). Moreover, beyond their industrial applications, EPS are increasingly recognized for their positive impact on human health (Ale et al., 2016; Zannini et al., 2018). QE fermentation was carried out individually with each strain and using a mixture of both. To induce the synthesis of EPS in *L. fermentum* Lf2, two concentrations of sucrose (2 and 4% w/v) were assayed. Although there were no significant effects on the total LAB counts (Figure 4) for the strains used as individual cultures, nor for the mix, a trend to lower pH (Figure 5) was observed as the concentration of sucrose increased. After 6 h of fermentation, QE inoculated with LpAv and the mix exhibited significantly lower pH values with 2% (w/v) compared to 4% (w/v) of sucrose. Additionally, only LpAv-QE reached a pH below 4.5 when 4% (w/v) of sucrose was used. After 8 h of fermentation with 4% (w/v) sucrose, all samples reached a pH of 4.5.

#### 3.4.2 Rheological analysis of fermented QE

**Figure 6:**
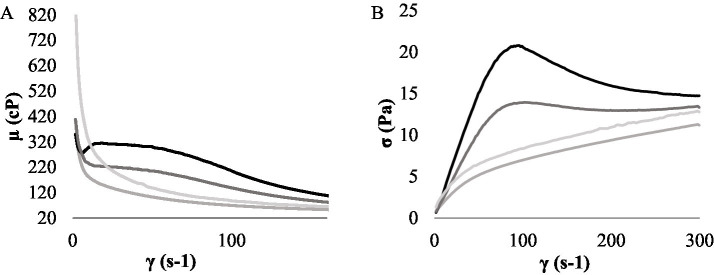
Apparent viscosity (*μ*) versus shear rate (*γ*) **(A)**, and shear stress (*σ*) versus γ **(B)** for the EQ with the addition of 4% (w/v) of sucrose, including the non-fermented control (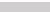) and samples fermented with *LpAv* (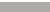), *Lf2* (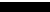), and the mix (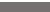). Values are means of three repetitions.

Due to sensory perception of changes in consistency and flavor characteristics, a sucrose concentration of 4% (w/v) was selected for further characterization of the protoype, as higher concentrations of the disaccharide were perceived as overly sweet and also contributed to a slower decrease in pH (data not shown). The rheological behavior of the QE-based product with the addition of 4% (w/v) sucrose was studied in both fermented (LpAv, Lf2, mix) and non-fermented forms. As shown in Figure 6A, viscosity decreased while the shear rate increased, a characteristic of pseudo-plastic fluids (Li et al., 2021). In general, the shear stress (Figure 6B) and viscosity values for QE fermented with Lf2 were higher than those of the other products, except at the end of the shear rate sweep (300 s^−1^ and 150 s^−1^), where Lf2-QE was only significantly higher than LpAv-QE (*p* < 0.05). The behavior of LpAv-QE was similar to that of the non-fermented control throughout the shear rate range (*p* > 0.05). The shear stress and viscosity values of the mix sample showed an intermediate behavior between LpAv-QE and Lf2-QE, being higher than those of the non-fermented control and LpAv-QE up to 150 s^−1^ (*p* < 0.05), then leveling off to values comparable to the control and LpAv toward the end of the sweep (*p* > 0.05).

#### 3.4.3 Chemical determinations

The concentration of sucrose, lactic acid and ethanol in non-fermented and fermented QE are shown in Table 2. Sucrose concentration in fermented samples were significantly lower (*p* < 0.05) compared to non-fermented QE. Moreover, a significantly higher consumption (*p* < 0.05) of sucrose was observed in the Lf2-QE samples compared to LpAv-QE, which may be attributed to the utilization of sucrose by Lf2 for the synthesis of EPS. On the other hand, the mixed culture produced the highest concentration of lactic acid, showing statistically significant differences compared to the other fermented samples. Ethanol was only detected in Lf2-QE and Mix-QE, with concentrations of 0.30 ± 0.01 and 0.26 ± 0.02 g/L, respectively.

**Table 2:** Sucrose, lactic acid and ethanol (g/l) in non-fermented quinoa extract (QE) and in QE fermented with *L. plantarum* LpAv (QE-LpAv), *L. fermentum* Lf2 (QE-Lf2) or the mixture of both strains (QE-Mix).

Parameter	QE	QE-LpAv	QE-Lf2	QE-Mix
Sucrose	38.94 ± 0.21^a^	37.88 ± 0.40^b^	36.39 ± 0.09^c^	36.97 ± 0.48^b,c^
Lactic acid	Nd	1.50 ± 0.02^a^	1.14 ± 0.01^b^	1,61 ± 0,04^c^
Ethanol	Nd	Nd	0.30 ± 0.01^a^	0,26 ± 0,02^b^

^a,b,c^Values with different superscripts differ significantly (*p* < 0.05) for each sample. Values are means (±SD) three replicates. Nd, not detected.

#### 3.4.4 Peptidic profile

**Figure 7:**
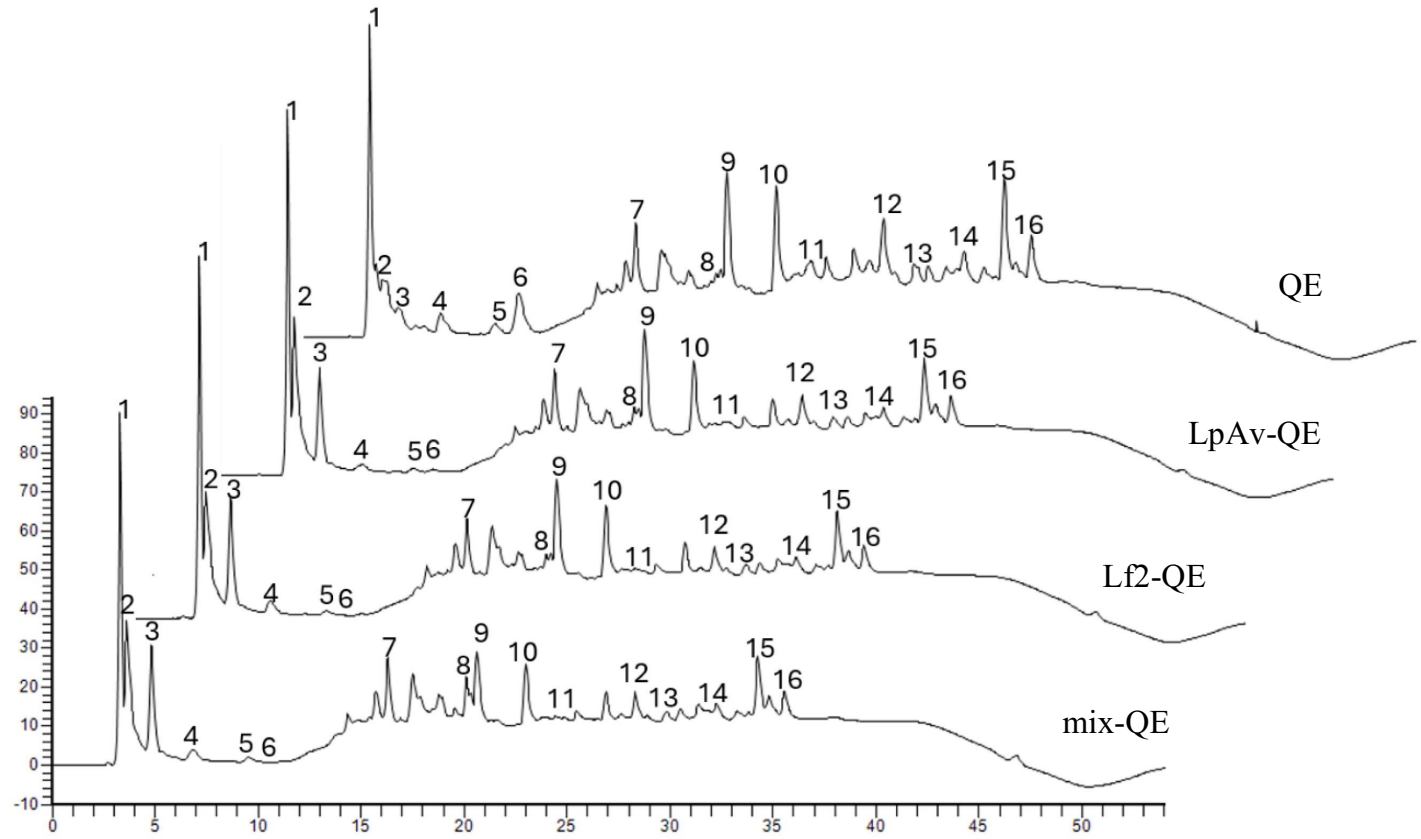
Peptide profiles determined by RP-HPLC of QE-LpAv, QE-Lf2, QE-mix and non-fermented QE. The numbers in the chromatogram indicate the peaks selected.

Chromatographic profiles of soluble peptides in fermented and non-fermented QE are shown in Figure 7. Sixteen peptides that exhibited the most notable changes, either increases or decreases in peak areas, are indicated in the figure. In the profiles of non-fermented QE (NF), the peak areas between 6 and 17 min (peaks 4, 5, 6, and 7), as well as those between 20 and 36 min (peaks 9 to 16), were higher than those observed in the fermented LpAv-QE, Lf2-QE, and Mix-QE. No marked differences were noted for these peaks among the fermented samples. On the other hand, the peak areas between 3 and 5 min (peaks 1, 2, and 3) were higher in the fermented QE than in the NF. Additionally, the peak 8, observed at 20 min, was higher in the mix-QE than in either LpAv-QE or Lf2-QE, as well as the non-fermented.

#### 3.4.5 Survival of LAB in fermented QE during storage

**Figure 8:**
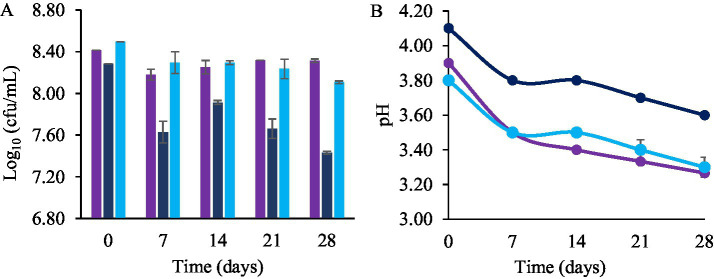
Total LAB count (Log_10_ cfu/mL) (A) and pH values (B) of QE fermented by *L. plantarum* LpAv (■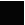), *L. fermentum* Lf2 (■), and mix (LpAv + Lf2) (■), at the beginning (0) and after 7, 14, 21 and 28 days of storage at 4°C. Values are means (±SD) of three repetitions.

The counts of *L. plantarum* LpAv and *L. fermentum* Lf2 in fermented QE were evaluated during refrigerated storage (4 °C) for 4 weeks (Figure 8). The bacterial counts at the beginning of the storage period were higher than 8 log cfu/mL in all fermented samples. The levels of *L. plantarum* LpAv remained stable throughout the storage, decreasing from 8.41 to 8.31 log_10_ cfu/mL after 28 days, without significant differences. Although *L. fermentum* Lf2 counts decreased by 0.7 log units after 7 days and by 0.85 log units by the end of the study, the difference between days 1 and 7 was not statistically significant. Regarding the Mix-QE, total LAB counts significantly decreased from 8.49 ± 0.10 to 8.29 ± 0.01 log orders after 7 days, with no significant differences observed when comparing 14, 21 and 28 days. Despite reductions in viability, the final concentration of LAB remained above 7 log cfu/mL in LpAv-QE and Mix-QE. The pH values of the fermented samples during storage (Figure 8) displayed a significant reduction (*p* < 0.05) at 7 days with stable values observed until the end of the assay.

#### 3.4.6 Sensorial analysis

A total of 111 consumers participated in the test, ranging from 18 to 50 years old, including Faculty students and staff members from the Faculty of Chemical Engineering, with a 54.5% of female participants. The overall acceptability, expressed as a weighted mean, of the fermented QE samples with strawberry and vanilla flavors was 4.8 and 4.7, respectively. According to the scale used, these values correspond to “dislike slightly” and “neither like nor dislike.” However, when acceptability was analyzed by age group, a trend was observed in which participants aged 30 to 50 reported higher acceptability, with weighted means of 6.0 and 5.5 for the strawberry- and vanilla-flavored QE, respectively (Table 3). Furthermore, 22.52% of participants rated the strawberry-flavored fermented QE with a hedonic score of 7 or higher, whereas this percentage decreased to 16% for the vanilla-flavored sample. Overall acceptance scores for the fermented quinoa beverages were low, falling within the “dislike slightly” range across treatments. Beyond flavor and color, panelists frequently reported intrinsic sensory characteristics associated with the fermented quinoa extract. These included detectable bitterness, slight astringency, and a dry or coarse mouthfeel. In several cases, these attributes persisted despite the addition of artificial flavorings, indicating that flavor masking did not fully offset the underlying sensory properties of the fermented matrix. Sourness associated with fermentation was also noted in some evaluations and contributed to the overall perception of the beverages. Together, these factors appear to have influenced the low acceptance ratings observed.

**Table 3:** Weighted averages (WA) for the fermented quinoa extract with strawberry (A) or vanilla (B) flavor, for three age ranges.

Flavor	General WA	WA ages 13–20	WA ages 21–30	WA ages 13–20
A	4.77 ± 0.21	4.30 ± 0.35^a^	5.31 ± 0.24^b^	6.05 ± 0.50^b^
B	4.70 ± 0.08	4.28 ± 0.27^a^	5.00 ± 0.21^a^	5.52 ± 0.45^a^

^a,b^Values with different superscripts in each column corresponding to the three age ranges differ significantly (*p* < 0.05). Values are WA ± standard deviations (SD) from participant evaluations.

### 3.5 Animal trial

#### 3.5.1 Translocation and immunomodulation potential

**Figure 9:**
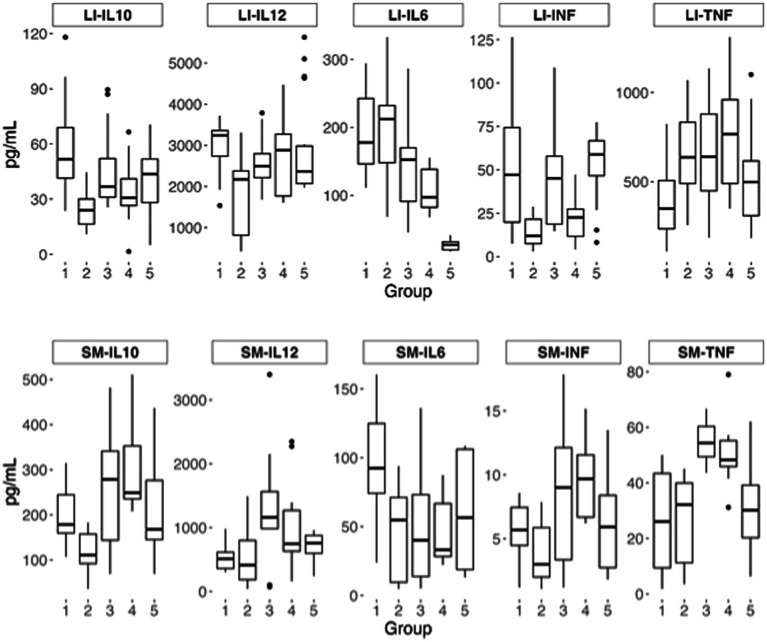
Levels of the cytokines IL10, IL12, IL6, INFγ, and TNFα. Mice that received *LpAv*-QE (1), *Lf2*-QE (2), mix-QE (3), and controls with non-fermented quinoa (4) and water (5).

No bacterial colonies were observed on VRLB agar plates inoculated with liver homogenates, indicating the absence of bacterial translocation. The immunomodulatory potential was assessed both in the small intestine (intestinal fluid and tissue homogenate) and large intestine (tissue homogenate) (Figure 9). Regarding the levels of S-IgA in the small intestine fluid, no significant differences were observed among groups. Values observed were 15.3 ± 4.3 ng/mL, 11.8 ± 2.7 ng/mL, 11.0 ± 5.9 ng/mL, 10.7 ± 4.1 ng/mL and 16.2 ± 2.4 ng/mL for QE fermented with LpAv, Lf2, the mix and the controls not fermented QE and tap water, respectively. In homogenates of the small intestine, the levels of IL-6 and IL-12 showed no significant differences among the treatments, while the concentrations of the pro-inflammatory cytokines INF-*γ* and TNF-*α* were significantly higher in animals that received LpAv-QE and Lf2-QE compared to controls. Additionally, LpAv-QE and Lf2-QE treatments significantly increased the levels of IL-10 compared to the control group that received non-fermented QE. In the large intestine, the concentration of IL-10 significantly increased in the LpAv-QE group compared to non-fermented control, and Lf2-QE significantly decreased the IL-10 levels compared to water control. The lowest concentration of IL-6 was found in mix-QE treatment, compared to all the other groups, while IL-6 in the Lf2-QE group was significantly lower compared to controls and LpAv-QE. The level of INF-γ was significantly increased in groups LpAv-QE and mix-QE compared to Lf2-QE and in the non-fermented control.

#### 3.5.2 Oxidative stress and enzyme tissue damage in liver

**Figure 10:**
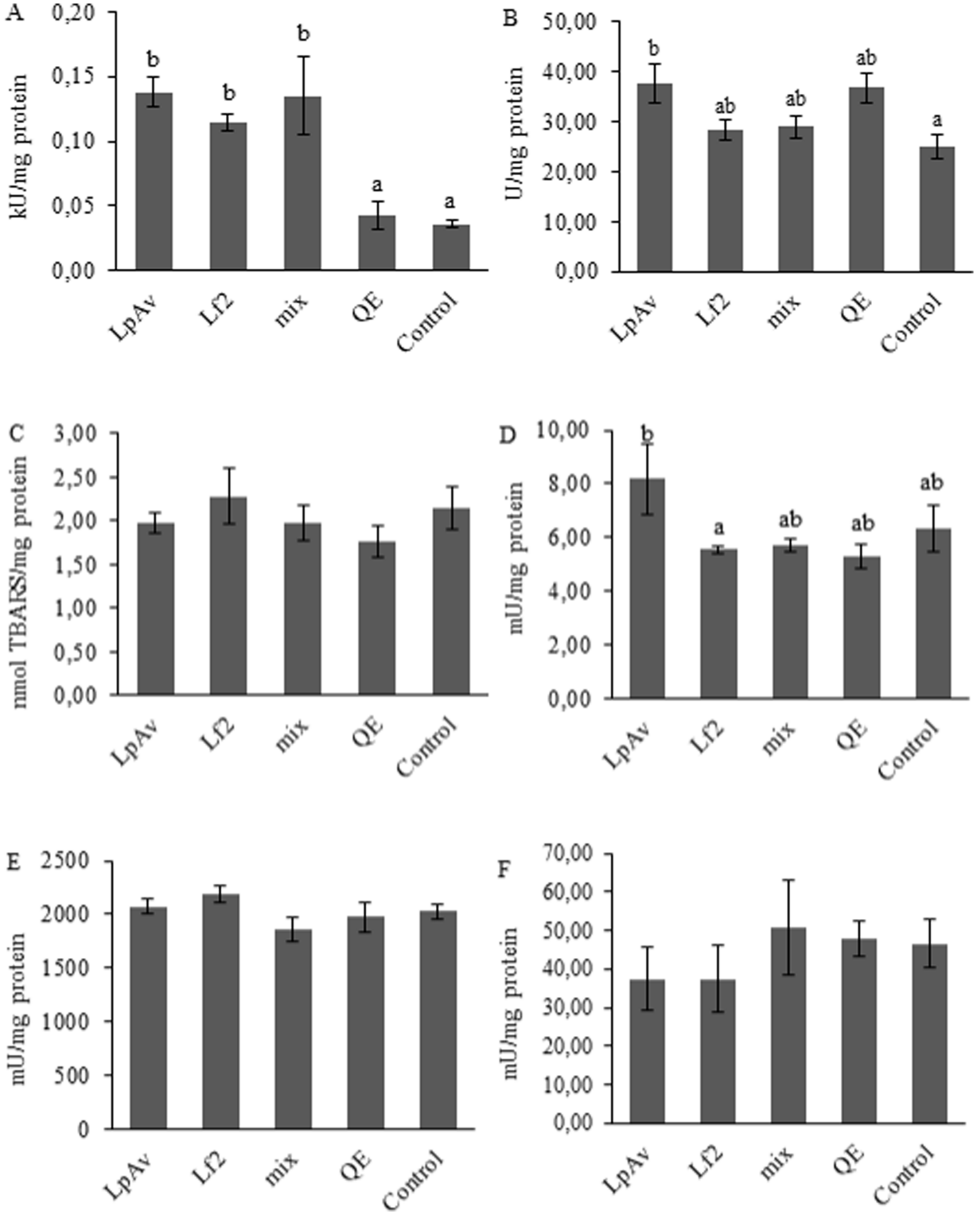
Enzyme activity and lipid peroxidation levels in liver. CAT **(A)**, SOD **(B)**, LPO **(C)**, FA **(D)**, GPT **(E)**, and GOT **(F)**. Mice that received LpAv-QE, Lf2-QE, mix-QE, and controls with non-fermented quinoa extract and water (control). Results are expressed as ®*x* ± SEM. Different letters represent significant differences (*p* < 0.05) between treatments.

The activity of CAT in the liver (Figure 10) significantly increased in all the groups that received fermented QE when compared to both controls (non-fermented and water). The SOD activity was significantly higher in the LpAv-QE group compared to water control. Regarding the LPO levels, no differences were found among the treatments. In terms of enzyme tissue damage, only ALP activity showed significant differences in LpAv-QE group compared to Lf2-QE treatment (whereas no differences were observed for GPT or GOT enzyme activities).

#### 3.5.3 Determination of organic acids in feces

**Figure 11:**
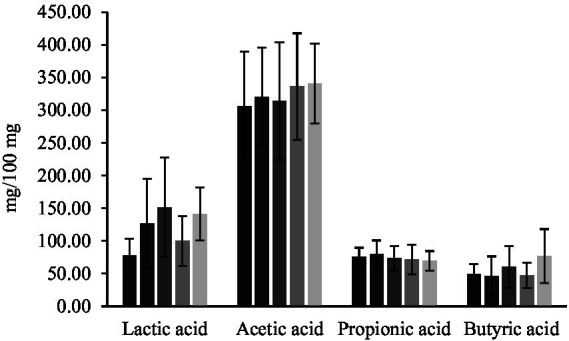
Concentration of organic acids in feces. Lactic acid, acetic acid, propionic acid, and butyric acid in mice that received *LpAv*-QE (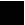), *Lf2*-QE (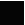), mix-QE (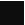), QE (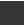), and control (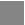). Values are means (±SD).

The concentrations of short-chain fatty acids are displayed in Figure 11. There were no significant differences in the levels of acetic, propionic, butyric and lactic acid among the experimental groups.

## 4 Discussion

### 4.1 Development of freeze-dried cultures

In this work we aimed at developing a prototype of a fermented beverage of quinoa, using LAB strains isolated from plant material (Puntillo et al., 2020) and from cheese (Ale et al., 2016) in our laboratory. The development of functional foods using locally sourced strains is of special interest in emerging countries as it promotes local research and development activities and people, in general, confer special credibility and value to the development of local foods (Sybesma et al., 2015). When aiming at obtaining a plant-based fermented product, a crucial consideration is the composition of the culture medium used for the production of the starter cultures. MRS is a world-wide known and used culture medium specifically designed to optimize the growth of strains of the genus formerly known as *Lactobacillus*, its commercial formulation includes animal-derived components such as meat extract or casein peptone (De Man et al., 1960). Then, a plant-based version of MRS (MRSv) was formulated *in house* by replacing the meat peptone in commercial MRS by soy peptone. Although *μ*_max_ values in MRSv were lower compared to MRS for most strains, similar DO_570nm_ values after 10 h of incubation indicated that the animal-free medium MRSv yield satisfactory growth of the LAB strains under study. Similar trends have been previously reported, with strain-dependent variations in growth performance depending on the nitrogen source employed (Garrote Achou et al., 2025).

Freeze-drying is the technology most used for the preservation of LAB cultures, it involves freezing and sublimation, two harsh processes that may present challenges for bacterial survival. To increase the survival rate, protectants are usually added before lyophilization, with carbohydrates being the most common type of cryoprotectants (Cui et al., 2022). Maltodextrin is a partially hydrolyzed starch ingredient widely used as drying agent due to its effectiveness during drying, minimal crystallization during storage, low cost, and mild flavor (Barbosa et al., 2015). While there is a substantial body of research on microbial drying and preservation, resistance remains strain specific (Morgan et al., 2006; Wang et al., 2021). The differences in survival along storage observed in this work highlights the importance of assessing the survival of each strain under consideration.

During the storage of freeze-dried cultures, bacterial cells in dehydrated format are exposed to various stress factors, including oxidation by air exposure and temperature, which affects shelf-life stability during storage (Senovieski et al., 2024). In this work, two storage temperatures were studied. When compared, refrigeration at 4 °C effectively maintained higher levels of viable cells (>9 log cfu/g) for the freeze-dried cultures (except for *L. plantarum* LpAv) over 12 months, in contrast to storage at 22 °C which resulted in lower levels (<9 log cfu/g). This aligns with observations widely reported in scientific literature that the shelf-life of freeze-dried bacterial products is highly dependent on storage temperature, with higher temperatures leading to lower viability rates (Morgan et al., 2006). The results obtained suggest that *L. plantarum* LpAv is the most resistant strain among the ones studied and has the highest potential to be further developed and used as starter culture. If other strains are of interest, further work should be conducted to improve their survival capacity along storage as freeze-dried cultures. Technological solutions to improve survival to freeze drying described for other LAB include optimizing the growth phase at harvesting, to apply mild heat or osmotic stress before freeze drying to study the impact of different cryoprotectants such as skim milk, trehalose, or sucrose, and to apply microencapsulation techniques to physically shield cells during drying and storage. Adjusting the residual moisture content and packaging atmosphere can also markedly enhance stability (Carvalho et al., 2004). Implementing one or a combination of these strategies, particularly the use of protective carbohydrates and encapsulation matrices, could substantially increase the viability of *L. fermentum* Lf2 during commercial freeze-drying processes.

### 4.2 Development of a prototype of fermented quinoa beverage

Along with potential health benefits, plant-based material may also contain heat-resistant opportunistic pathogens which may compromise the safety of the product (Soni et al., 2022). Lactic fermentation produces organic acids, hydrogen peroxide and bacteriocins, which may further contribute to the inhibition of spoilage microorganisms and foodborne pathogens (Mokoena et al., 2021). A fast reduction of pH during fermentation to values below 4.5 is crucial for improved microbiological safety of the fermented product, as this threshold inhibits the growth of potential pathogens (Liu et al., 2023). For instance, fermenting soymilk with either *L. plantarum* Tennozu-SU2 or *L. lactis* BF1 effectively prevented contamination by *Salmonella typhimurium* and *Listeria monocytogenes* both before and after the fermentation process during storage (Haraguchi et al., 2019). In another study, *Enterobacteriaceae* was found in heat-treated (pasteurized) unfermented quinoa beverage, but fermentation with *L. plantarum* DSM 9843 effectively reduced *Enterobacteriaceae* levels to below detectable limits (Canaviri Paz et al., 2020). In this work, and except for *L. rhamnosus* LrM1 and *L. paracasei* LpaM1, all the other strains displayed satisfactory capacity to reduce pH to values lower than 4.5.

Counts of LAB after QE fermentation achieved concentrations higher than 8 log orders cfu/mL except for *L. paracasei* LpaM1. Similar counts were reported by Karovičová et al. (2020) in a quinoa beverage using a commercial starter culture containing *Bifidobacterium* sp., *L. acidophilus*, and *S. thermophilus*. Similarly, Cerdá-Bernad et al. (2022) obtained counts of 8.20 log_10_ cfu/mL for QE fermented for 12 h with *L. plantarum* CECT 220. Conversely, other scientific publications reported higher counts of LAB, such as 9.5 log cfu/mL after of 6 h of QE fermentation but initiating fermentation with an inoculum of ca. 8 log cfu/mL (Ludena Urquizo et al., 2017). Dallagnol et al. (2013) studied the fermentative and proteolytic activity of *L. plantarum* CRL 778 in the fermentation of quinoa and Wheat slurries, finding a maximum count of 9.8 log cfu/mL. The fermentation time in plant-based beverages depends on several factors, including the substrate type, the fermentation conditions and the starter culture intrinsic properties, with the latter playing a pivotal role in achieving the desired product outcome (Yang et al., 2024). The evaluation of the capacity of the starter cultures to ferment non-dairy substrates is a crucial step in the development of new fermented products. In this work, we confirmed that quinoa is a suitable substrate for lactic acid fermentation if the proper LAB strains are available, highlighting the differences in performance among strains (strain-dependency) and the importance of selecting an appropriate starter culture. Based on the higher rate of pH decrease and LAB counts obtained, *L. plantarum* LpAv and *L. fermentum* Lf2 were selected to continue with the development of a prototype of a fermented quinoa beverage. *L. plantarum* LpAv displayed immunomodulatory potential in previous work (Puntillo et al., 2021). *L. fermentum* Lf2 is an EPS producing strain (Ale et al., 2016), which could improve the sensorial characteristics of the fermented product (Zannini et al., 2018). In a previous study, yogurts made with *L. fermentum* Lf2 strain, along with the starter culture, increased the release of bioactive peptides in the product and had a significant positive impact on the levels of organic acids, carbohydrates, the rheological properties and the microstructure (Ale et al., 2023). Furthermore, beyond the technological and functional relevance of EPS and their industrial applications, there is growing evidence that these compounds may exert a positive impact on human health by acting like prebiotics (Ale et al., 2016).

The use of *L. plantarum* in the fermentation of plant-based products, particularly quinoa, has been described in various studies, yielding products with diverse characteristics and reaching suitable pH values at different fermentation times. Ayub et al. (2021) reported the production of fermented quinoa with reduced phytates content through fermentation with *L. plantarum* 299v. Fermentation of quinoa with *L. plantarum* CRL 778 increased the amount of free amino acids, enhancing their bioavailability (Dallagnol et al., 2013). Quinoa fermented with *L. plantarum* T6B10 exhibited increased concentrations of free amino acids, *γ*-aminobutyric acid, polyphenols, antioxidant activity, and protein digestibility (Lorusso et al., 2018). *L. plantarum* LpAv showed the ability to ferment the QE rapidly decreasing the pH values to desirable levels for the microbiological safety of the product. Kehinde et al. (2024) used *L. fermentum* MTCC 903, a LAB strain with high proteolytic activity, to ferment quinoa. Their study, focused on proteolytic activity, antinutrient reduction, and increased antioxidant power, observed a pH of 3.2 after 24 h, demonstrating the fermentative of *L. fermentum* MTCC 903 to ferment quinoa. Additionally, quinoa fermented with *L. fermentum* PTCC 1638 resulted in pH value of 4.5 after 18 h of fermentation but using an initial inoculum of 5.2–5.4 cfu/mL, approximately (Jafarpour and Hashemi, 2023).

The use of exopolysaccharide EPS-producing LAB as starter cultures has emerged as a promising strategy to enhance the rheological properties of plant-based yogurt-like products (Montemurro et al., 2021). In-situ EPS production improves viscosity and contributes to the formation of network-like gel structures (Xu et al., 2018). Beyond sensory improvements, EPS offer health benefits due to their immunomodulatory properties, linked to their complex chemical structures (Wang et al., 2025). In this context, the rheological behavior observed in QE fermented with Lf2 and the mix could be attributed to the production of exopolysaccharides by *L. fermentum* Lf2. The increase in viscosity observed in the QE fermented with the mix and Lf2 compared to the non-fermented control is consistent with reports from various authors for QE fermented with EPS-producing strains (Bianchi et al., 2015; Karovičová et al., 2020; Lorusso et al., 2018). Moreover, Zannini et al. (2018) reported that fermentation of QE with the EPS-producing strain *Weissella cibaria* MG1 resulted in a product with significantly higher apparent viscosity (*p* < 0.05) compared to the non-fermented control, due to the levels of EPS produced. To the best of our knowledge, studies on the fermentation of quinoa with EPS-producing *L. fermentum* strains have not been reported. However, some works studied the fermentation of quinoa with EPS-producing LAB, mainly *Weissella* strains. Zannini et al. (2018) obtained a product with high water-holding capacity, preventing syneresis, higher viscosity (0.57 mPas), and a high EPS concentration (40 mg/L) using *W. cibaria* MG1 and 10% of sucrose, compared to a chemically acidified unfermented control. However, the pH value was 5.16. On the other hand, *W. confusa* DSM 20194 was used in another study to ferment quinoa supplemented with 10% sucrose, resulting in increased viscosity, reduced syneresis, and a pH of 5.1 after 20 h of fermentation (Lorusso et al., 2018).

As mentioned previously, during the fermentation process compounds are produced as a result of the metabolism of substrate components by LAB. The identification and quantification of these metabolites is of interest for the development of new products. The levels of lactic acid detected in this study were higher than those reported by Karovičová et al. (2020) and Cerdá-Bernad et al. (2022), who obtained 0.46 ± 0.02 and 0.36 ± 0.01 g/L, respectively, after 6 h of QE fermentation. Conversely, these values were lower than those reported by Hurtado-Murillo et al. (2024), who fermented a substrate containing 90% of quinoa flour and obtained a lactic acid concentration of 1.9 g/L. Additionally, ethanol was detected in the QE-Lf2 and the QE-mixed, which can be attributed to the fact that Lf2 is an obligate heterofermentative LAB. No lactic acid or ethanol was detected in the non-fermented control sample.

The differences in peak areas in the peptidic profiles among samples suggest that inoculation with LpAv and Lf2, either individually or in combination (mix), resulted in an overall increase in peptidase activity. This outcome is consistent with previous findings, as the positive contribution of *Lactobacillus* species to proteolysis and peptidolysis in fermented foods has been widely documented across various matrices (Dallagnol et al., 2013; Gimenez et al., 2024; Peralta et al., 2020, 2023; Satılmış et al., 2023). Microbial peptidases are well recognized for their critical role in cheese ripening (Ardö, 2021), especially in contributing to the flavor by breaking down large peptides, including some with bitter attributes, and by increasing the concentration of free amino acids that serve as flavor precursors (Zhao et al., 2016). Furthermore, their positive effects were reported in plant-based foods (Jakubczyk et al., 2013; Rizzello et al., 2017). Regarding quinoa, Dallagnol et al. (2013) reported about proteolytic activity of *Lactobacillus plantarum* CRL 778 in quinoa and wheat slurries. In particular, the contribution of Lf2 to peptidolysis has been previously demonstrated both *in vitro* and *in situ* in yogurt (Ale et al., 2023). On the other hand, LpAv has been reported to successfully ferment oat and carrots substrates. Moreover, the administration of fermented carrots to BALB/c mice conferred an increase in survival (80%) of *Salmonella*-infected mice compared to the control group (<25%) (Puntillo et al., 2021).

The biggest area of the peak 8 in mix-QE suggests a potential synergistic effect between *L. plantarum* LpAv and *L. fermentum* Lf2 in the production of this specific peptide. Cooperation among different strains has been extensively studied for various purposes, including improved fermentation performance and enhanced flavor development (Amárita et al., 2006; Ayad et al., 2001; Kieronczyk et al., 2003). For example, a well know cooperation is the mutual growth stimulation between *S. thermophilus* and *L. delbrueckii* ssp. *bulgaricus* in yogurt (Smid and Lacroix, 2013). In general, under the conditions used for the peptide profiling analysis, the more hydrophobic peptides, typically eluting later, are associated with bitter sensory attributes (Peralta et al., 2024). Given that fermented QE exhibited lower levels of hydrophobic peptides, it could be hypothesized that the fermentation process contributes to improved sensory properties. Nevertheless, further studies are necessary to validate this hypothesis.

The viability of LpAv after a four-week storage was consistent with previous studies involving *L. plantarum* strain (Lorusso et al., 2018; Ludena Urquizo et al., 2017). This loss in LAB viability may be attributed to the strain’s sensitivity to cellular damage caused by prolonged exposure to the acidity of the fermented product (Soto et al., 2023). However, resistance depends on many factors, such as the specific strains and the substrates. The concentration of *L. plantarum* Q823 reported by Väkeväinen et al. (2020), was higher than 9 log cfu/mL after 28 days of storage. These findings are consistent with those reported by Canaviri Paz et al. (2020), who obtained a final pH of 3.61 after 28 days of storage in a quinoa-based beverage fermented with *L. plantarum* DSM9843. Similarly, Ludena Urquizo et al. (2017) and Dallagnol et al. (2013) also reported significant pH reductions over the same storage period in beverages fermented from QE. This phenomenon of post-acidification, in which pH values decrease during refrigerated storage, is due to continued metabolism of residual sugars in the substrate by LAB, resulting in lactic acid production (Deshwal et al., 2021). However, Lorusso et al. (2018) noted that, despite a pH decrease to 3.6 during 20 days of storage at 4 °C, the fermented product was still sensorially acceptable, aligning with previous studies that demonstrate high sensory stability in plant-based beverages fermented with LAB.

In addition to fermentation, flavoring agents play an important role in enhancing acceptability. Ramos Chilo (2020) and Karovičová et al. (2020) reported a higher preference for fruit-flavored fermented beverages, such as those containing pineapple or raspberry. Similarly, Ludena Urquizo et al. (2017) observed an increase of 3.3 points on the hedonic scale when fermented QE was flavored with blueberry or chocolate. For this reason, in the present study, strawberry and vanilla essences were added to the fermented QE to improve its acceptability. Additionally, a pink colorant was incorporated into the strawberry-flavored sample to align visual perception with the expected flavor.

A mean hedonic score of 7 or higher on a 9-point scale is commonly interpreted as an indicator of high sensory acceptability (Everitt, 2009). In this work, overall acceptability values below this threshold may be attributed to the intrinsic sensory characteristics of fermented QE, despite the addition of flavorings. Although some plant-derived components, such as soluble fibers, can improve texture and mouthfeel, plant-based alternatives to milk are often perceived as less pleasant, possibly due to prior unfavorable experiences with such products (Tangyu et al., 2019). Recent studies in adults suggest that dietary patterns rich in plant-based foods may promote overall healthy aging. Accordingly, adults value nutritional quality and incorporate fruits, vegetables, whole grains, unsaturated fats, nuts, legumes, and other nutrient-dense foods into their diets (Tessier et al., 2025).

It is important to note that consumer acceptance is not determined solely by sensory properties. Demographic factors, including age, cultural background, and dietary habits, significantly influence sensory preferences and liking scores (Torrico et al., 2019). Moreover, consumers’ beliefs and perceptions about food play a central role in their consumption and purchasing decisions. These perceptions are shaped by product familiarity, demographic characteristics (e.g., nationality, gender, age), and the cultural context of consumption (Ares et al., 2008). Food culture developed during childhood and continuous exposure to food-related information also influence knowledge and attitudes toward foods (Johansen et al., 2011). Although the modest acceptance scores suggest that intrinsic sensory properties of the fermented quinoa extract—such as bitterness, astringency, or mouthfeel characteristics—may have influenced consumer perception, this interpretation should be regarded as a working hypothesis. The present study did not include a focused sensory evaluation designed to identify the specific attributes that drive liking or disliking of the beverages. Therefore, additional sensory validation is needed to confirm whether these proposed attributes indeed underpin the low acceptance observed. Future studies employing descriptive sensory profiling, consumer mapping, or targeted attribute diagnostics will be essential to substantiate this hypothesis and to guide formulation strategies aimed at improving the organoleptic quality of fermented quinoa beverages.

### 4.3 *In vivo* assessment of the functional potential of the fermented quinoa beverage

Mice have been a long term used model to explore the immune response observed after the oral administration of LAB or their fermented beverages, with a great range of outcomes observed among studies which can be explained by the fact that the response depends on the specific strain/s administered and the food matrix used for fermentation (milk, meat, vegetables, fruits, and cereals). Many authors agree that the induction of the anti-inflammatory cytokine IL-10 is an outcome of interest, however opinions are not consistent when it comes to proinflammatory cytokines such as TNF-*α*, IL-6, and IL-12. Karamese et al. (2016) studied the effect of a mix of *Lactobacillus* and *Bifidobacterium* strains in rats and found an increase in IL-10 expression, important for maintaining the integrity and balance of epithelial tissues (Iyer and Cheng, 2012), along with a decrease in TNF-α and IL-6. On the other hand, Morita et al. (2002) and Perdigón et al. (2002) evaluated different LAB strains and reported increases in both proinflammatory cytokines (IL-6, IL-12, TNF-α, and IFN-*γ*) and regulatory cytokines (IL-10 and IL-4). In this study, the absence of a clearly polarized response (toward an inflammatory or anti-inflammatory profile), along with the lack of significant differences in s-IgA levels, suggests that fermented QE primarily modulated the innate immune response. The increase in pro-inflammatory cytokines observed in our study can be further discussed. It is well established that several LAB strains can transiently activate components of the innate immune system, leading to moderate elevations in cytokines such as TNF-α, IL-6, or IFN-γ without inducing tissue damage or a pathological inflammatory state. Rather than representing detrimental inflammation, these responses often reflect immune stimulation and improved readiness of the mucosal immune system (Perdigón et al., 2002). The simultaneous increase in IL-10 in mice receiving fermented QE further supports this interpretation, as balanced induction of regulatory and pro-inflammatory mediators is characteristic of a controlled, strain-dependent immunomodulatory effect aimed at maintaining intestinal homeostasis (Iyer and Cheng, 2012). Thus, the cytokine profile elicited by LpAv-QE and Lf2-QE appears to represent a moderate and coordinated activation of mucosal immunity rather than a shift toward harmful inflammation.

Some *Lactobacillus* strains have been reported to exhibit efficient antioxidant systems capable of protecting against oxidative stress and related chronic diseases (do Nascimento et al., 2022). In this study, only CAT activity was significantly enhanced in all groups receiving fermented QE compared to both controls (non-fermented and water). Moreover, the administration of *L. plantarum* LpAv-QE also enhanced SOD and FA activities in liver. Overall, the results indicate that the antioxidant effects of fermented QE were strain-dependent. A previous study using the same *L. fermentum* strain also reported an enhancement of CAT activity in mouse liver, further supporting its potential antioxidant capacity (Ale et al., 2025). Similarly, the protective effects observed in animal studies have validated the potential role of *L. plantarum* in mitigating oxidative stress-related disorders (Ferdous et al., 2025). Gao et al. (2013) reported that administration of *L. plantarum* FC225, a strain isolated from fermented cabbage, resulted in increased hepatic SOD activity in mice fed a high-fat diet (Gao et al., 2013).

In previous studies that evaluated the impact of LAB or fermented beverages, fecal organic acids production was interpreted by comparing concentrations at the beginning and end of the administration period. This longitudinal approach, used for example by Salazar et al. (2015) and by Ale et al. (2019, 2025), enabled the authors to discern whether the treatment induced increases or decreases in SCFAs over time in the same mice. In our study, fecal samples were only collected at the end of the intervention; thus, baseline values were not available for comparison. As a consequence, although acetic, propionic, butyric, and lactic acids were quantified in feces, we cannot determine whether the measured levels represent an increase, decrease, or maintenance relative to pre-treatment conditions. This constitutes a limitation of this study when comparing our results with those reports and should be considered when interpreting the metabolic effects of LpAv-QE and Lf2-QE. Future studies including pre-administration fecal sampling would help clarify the dynamic response of SCFA production to the fermented quinoa beverages.

It is of interest to note that the fermented beverages under study were able to deliver what can be considered “a high dose of Live Dietary Microbes.” The concept of “Live Dietary Microbes” is emerging in the field of Nutrition. In 2022, a classification system for defining and estimating dietary intake of live microbes in US adults and children was proposed (Marco et al., 2022). A year later, positive health outcomes associated with the intake of live microbes from foods, including fermented foods, were assessed using the NHANES database (Hill et al., 2023). Certainly this concept fueled the interest of the scientific community and between 2024 and 2025, more than 20 papers reported the association of the intake of “Live Dietary Microbes” through food with less constipation, osteoporosis, depressive symptoms, biological aging, mortality, sarcopenia, metabolic syndrome, frailty, periodontitis, cardiovascular diseases, insulin resistance, prediabetes, steatotic liver disease, chronic obstructive pulmonary disease, and enhanced bowel health (for specific references, search PubMed with the words “Live Dietary Microbes”).

Although both fermented quinoa beverages elicited measurable antioxidant and immunomodulatory effects, these outcomes should be interpreted as modest initial indications of functional potential. To transition *L. plantarum* Av and *L. fermentum* Lf2 from live dietary microbes to validated probiotics, a series of studies is required. First, mechanistic work should clarify the specific metabolites or cellular structures responsible for the observed biological effects, including their interaction with host immune and redox pathways. Second, dose–response experiments and longer-term administration studies are needed to establish the minimal effective dose. Third, comprehensive safety evaluations—including assessment of antibiotic resistance profiles and potential for translocation—must be completed according to current probiotic guidelines. Finally, controlled human intervention trials will be essential to confirm whether the physiological effects detected in mice translate to measurable health benefits in humans. Together, these steps provide a clear roadmap for advancing the strains toward probiotic designation and for supporting their potential incorporation into functional foods.

## 5 Conclusion

In this work, *L. plantarum* LpAv and *L. fermentum* Lf2 stepped forward among six locally sourced LAB strains as satisfactory strains for the development of a prototype of a fermented quinoa beverage. Both strains displayed convenient stability as freeze-dried cultures and during storage of the fermented beverage. Though with a modest functional capacity as observed in the animal model used, the fermented quinoa beverage may contribute to the emerging concept of “Live Dietary Microbes” while further studies are conducted to determine their potential as probiotics.

## Data Availability

The raw data supporting the conclusions of this article will be made available by the authors, without undue reservation.
